# A recombination bin-map identified a major QTL for resistance to Tomato Spotted Wilt Virus in peanut (*Arachis hypogaea*)

**DOI:** 10.1038/s41598-019-54747-1

**Published:** 2019-12-03

**Authors:** Gaurav Agarwal, Josh Clevenger, Sandip M. Kale, Hui Wang, Manish K. Pandey, Divya Choudhary, Mei Yuan, Xingjun Wang, Albert K. Culbreath, C. Corley Holbrook, Xin Liu, Rajeev K. Varshney, Baozhu Guo

**Affiliations:** 1 USDA-ARS, Crop Protection and Management Research Unit, Tifton, GA USA; 2 University of Georgia, Department of Plant Pathology, Tifton, GA USA; 3 International Crops Research Institute for the Semi-Arid Tropics (ICRISAT), Center of Excellence in Genomics & Systems Biology, Hyderabad, India; 4 Mars Wrigley Confectionery, Center for Applied Genetic technologies, Athens, GA USA; 5 University of Georgia, Center for Applied Genetic Technologies, Athens, GA USA; 6 Shandong Academy of Agricultural Sciences, Peanut Research Institute, Qingdao, China; 7 Shandong Academy of Agricultural Sciences, Biotechnology Research Center, Jinan, China; 8 USDA-ARS, Crop Genetics and Breeding Research Unit, Tifton, GA USA; 9 BGI-Shenzhen, Shenzhen, China; 10 Present Address: The Leibniz Institute of Plant Genetics and Crop Plant Research (IPK), Gatersleben, Germany

**Keywords:** Genetics, Molecular biology, Plant sciences

## Abstract

*Tomato spotted wilt virus* (TSWV) is a devastating disease to peanut growers in the South-eastern region of the United States. Newly released peanut cultivars in recent years are crucial as they have some levels of resistance to TSWV. One mapping population of recombinant inbred line (RIL) used in this study was derived from peanut lines of SunOleic 97R and NC94022. A whole genome re-sequencing approach was used to sequence these two parents and 140 RILs. A recombination bin-based genetic map was constructed, with 5,816 bins and 20 linkage groups covering a total length of 2004 cM. Using this map, we identified three QTLs which were colocalized on chromosome A01. One QTL had the largest effect of 36.51% to the phenotypic variation and encompassed 89.5 Kb genomic region. This genome region had a cluster of genes, which code for chitinases, strictosidine synthase-like, and NBS-LRR proteins. SNPs linked to this QTL were used to develop Kompetitive allele specific PCR **(**KASP) markers, and the validated KASP markers showed expected segregation of alleles coming from resistant and susceptible parents within the population. Therefore, this bin-map and QTL associated with TSWV resistance made it possible for functional gene mapping, map-based cloning, and marker-assisted breeding. This study identified the highest number of SNP makers and demonstrated recombination bin-based map for QTL identification in peanut. The chitinase gene clusters and NBS-LRR disease resistance genes in this region suggest the possible involvement in peanut resistance to TSWV.

## Introduction

Cultivated peanut, *Arachis hypogaea*, is an economically important legume and serve as a major source of protein and vegetable oil for human nutrition. However, its productivity is severely challenged by foliar diseases such as Tomato spotted wilt virus (TSWV). Spotted wilt in peanut is arthropod-borne caused by thrips, vector of TSWV representing Tospovirus genus. TSWV in peanut is a complex disease and even long-term insecticide applications do not result in reduction in incidence of disease. TSWV has a major impact in southeastern U.S. peanut growing areas including Georgia, Florida and Alabama. It had resulted in an annual loss of $ 12.3 million in U.S. from 1996 to 2006^1^. Chemical pesticide application increases cost to peanut growers for disease management^2^ in addition to environmental pollution. Therefore, it is imperative to understand the genetics of host resistance and to identify the markers and genes for breeding high resistant peanut cultivars to be used by growers in order to prevent the yield and economic loss.

Cultivated peanut is an allotetraploid in its genetic constitution with AABB genome architecture with A-genome inherited from *Arachis duranensis* and B-genome inherited from *Arachis ipaensis*^3–5^. Relatively short evolutionary history along with the hybridization barriers between the wild diploids and the tetraploid peanut results in cultivated peanut with narrow genetic bases^6^. In order to identify the genomic regions responsible for disease resistance, studies have been conducted with a limited number of available simple sequence repeat (SSR) markers for map construction and QTL identification^7–11^ and as a result underlying gene(s) related to QTLs could not be identified. Therefore, identification of a large set of markers for the construction of high-density genetic maps to fine map the QTLs is necessary.

Massive SNP discovery in peanut was possible due to availability of the diploid reference genomes, the diploid progenitors, *A. duranensis*^3,12^ and *A. ipaensis*^3^. As a result, significant progress has been made in developing genomic resources like the first SNP chip with highly informative SNPs for breeding applications^13,14^ and identification of SNPs in potential candidate genes for disease resistance^15^. Deploying the whole genome re-sequencing (WGRS) technology, SNPs were identified between the parents (SunOleic 97R and NC94022). These parental SNPs were used to genotype the whole genome re-sequenced RIL population to identify polymorphic SNPs within the population. Utility and power of SNPs has recently been demonstrated in peanut by developing the first high-density genetic map containing 8,869 SNP markers obtained from Tifrunner × GT-C20 for QTL study^15^. In another study, QTL-seq approach was used where resistant and susceptible bulks were sequenced to identify QTLs for later leaf spot^16^.

Next generation sequencing (NGS) technology has facilitated high throughput identification of genome-wide SNPs. However, the high number of markers becomes a limitation for linkage map and QTL mapping software to handle. In order to convert large dataset into reliable and usable form, an approach of bin mapping has been used in several crop plants^17–20^. In this approach, a parent dependent sliding window is used to identify true recombination breakpoints using the SNP genotyping data of the entire RIL population, and these recombination bins instead of SNPs are used to construct the genetic linkage maps. Using this approach, QTLs for tassel and ear architecture in a F_2_ maize population has been mapped^21^ and candidate genes for drought tolerance in QTL hot spot region in chickpea have also been identified^22^.

In this study, we report the development of the first bin map using SNPs obtained from WGRS data of the parents, SunOleic 97 R × NC94022, and 140 RILs. Parents were sequenced at greater depth to identify reliable SNPs. However, the RILs were sequenced at lower depth. Using this data, a total of 11,106 SNPs in the form of 5,816 bins were used for the construction of genetic bin-map. Utility of this bin map has resulted in identification of QTLs and candidate genes for disease resistance. Further, Kompetitive allele specific PCR **(**KASP) markers associated with the major QTLs were developed that could be used as diagnostic markers in molecular marker-assisted selection (MAS) applications in breeding programs.

## Results

### Phenotypic variation in disease severity

**Figure 1:**
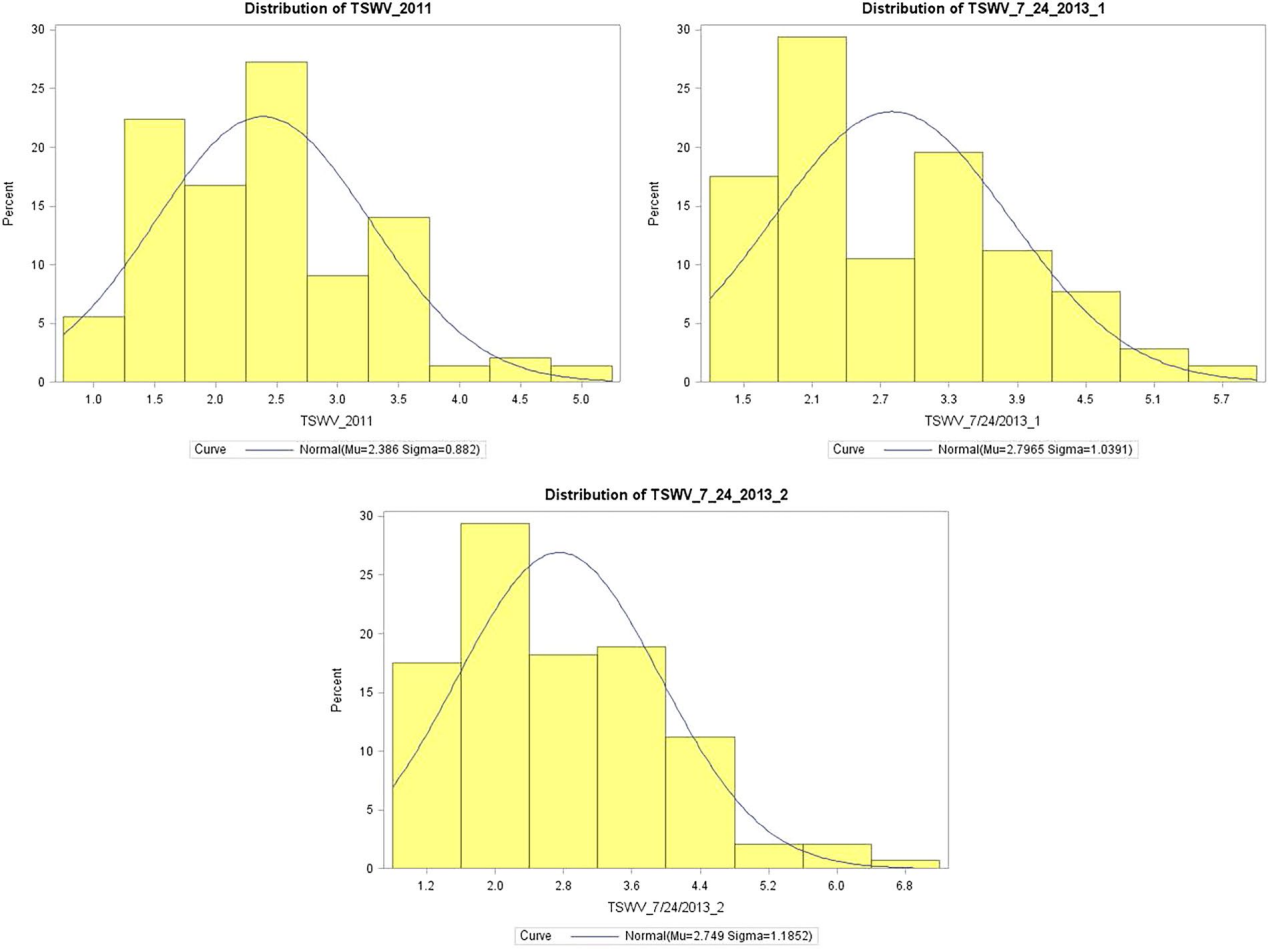
Phenotype distribution of disease ratings in SunOleic 97 R × NC 94022 RIL population. The y-axis represents the percentage of infected plants of Tomato spotted wilt virus (TSWV) in the population; the x-axis represents disease severity score on a scale of 1 (0% diseased) to 10 (91 to 100% diseased).

**Figure 2:**
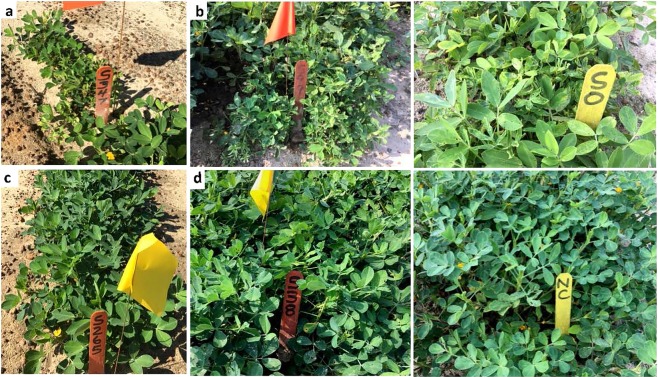
Resistant and susceptible RILs for TSWV in the early stage (**a,c**) and later stage (**b,d**), respectively. RILs showing extreme phenotypic variation for TSWV severity: the resistant lines S38 and S265, the susceptible lines S99 and S347 along with the parents, SO for SunOleic 97R and NC for NC94022.

A significant difference was observed among the RIL lines. The phenotypic variation of TSWV disease severity in these RILs was ranging from 1 to 7 in 2011 and 1 to 8 in 2013 (Supplementary Table S1). The phenotypic evaluation data showed a continuous distribution with a transgressive segregation (Fig. 1). The Shapiro-Wilk (w) test indicated that the distribution of the phenotypic data across multiple environments were normal in most of the seasons (Table 1). The ratings of disease severity for TSWV was distributed relatively normal except a few instances, in which some RIL lines of the population had extreme phenotypic ratings and were out of the normal range (Fig. 1). The RIL population in this study showed high phenotypic variability for TSWV. Some of the RILs showed high resistance and susceptibility to TSWV disease (Fig. 2).

**Table 1:** Descriptive statistical analysis for distribution of disease severity of Tomato spotted wilt virus (TSWV).

Trait_month_day_year	Kurt*	Skew*	w* (sig*)
TSWV_2011	0.205	0.595	0.955 (0.0001)
TSWV_7_24_2013	−0.049	0.706	0.942 (0.0001)
TSWV_7_24_2013_1	0.414	0.717	0.944 (0.0001)

*Kurt = kurtosis; Skew = skewness; w = Shapiro-Wilk statistic value; Sig = significance.

### Sequencing, SNP discovery and bin mapping

**Figure 3:**
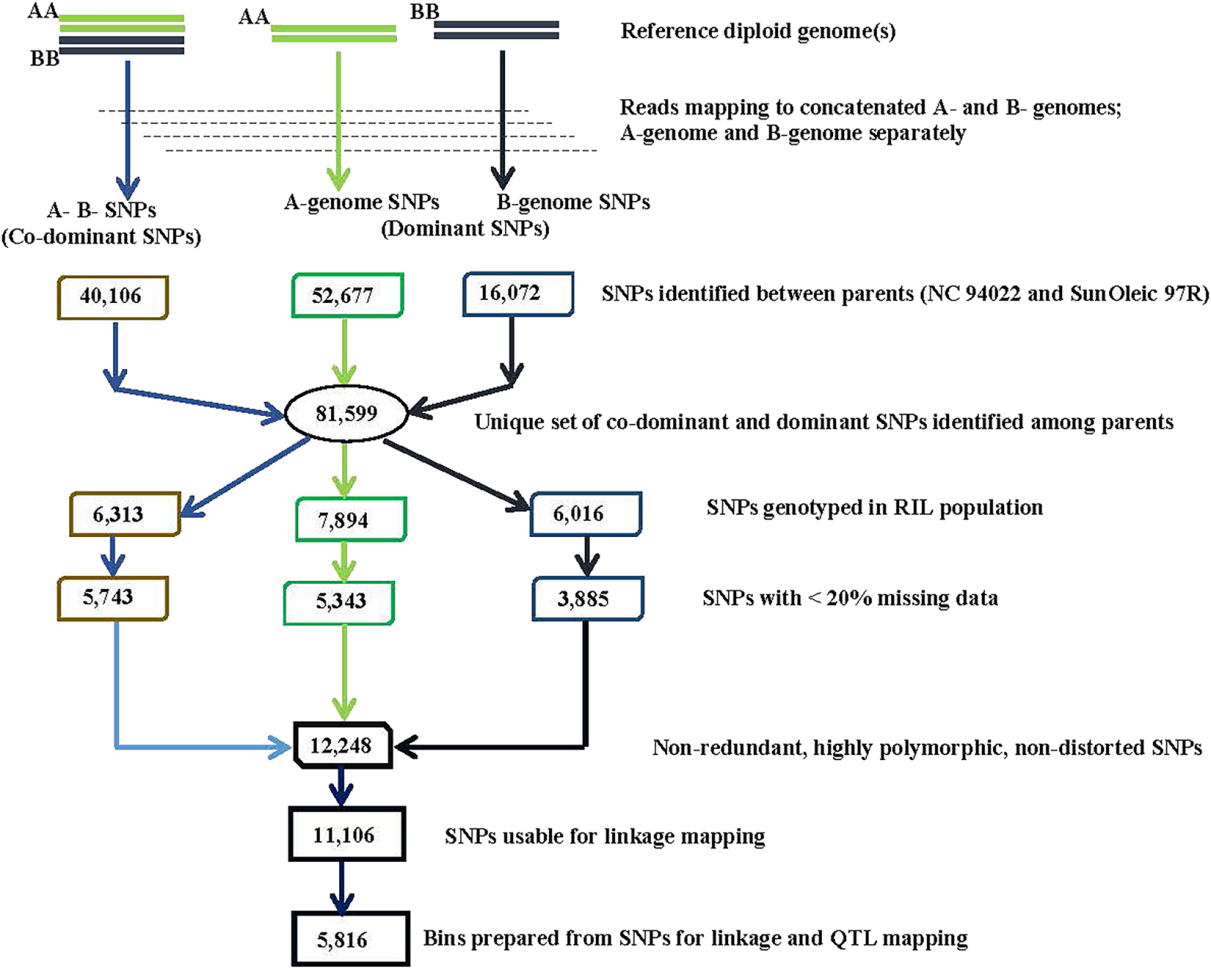
Workflow of identification of polymorphic co-dominant and dominant SNPs used for developing bin maps in peanut using whole genome re-sequencing data. Fastq files were aligned to the concatenated (A- and B-genome together) genomes and individually to A- and B-genomes in order to identify co-dominant and dominant SNPs. A-genome represents *Arachis duranensis* and B genome represents *Arachis ipaensis*. A total of 81,599 non-redundant SNPs was identified between the two parents using the above-mentioned approach. Individually, the co-dominant and dominant SNPs identified were used for genotyping the RIL population to identify polymorphic SNPs (alleles) present in the population. After removing the SNPs with more than 20% missing data, a total of 12,248 unique set of SNPs were obtained. Excluding the SNPs showing segregation distortion, a final set of 11,106 SNPs were used for identification of 5,816 bins used in construction of linkage maps.

A total of 1.8 Tb of data were generated for the parental lines, SunOleic 97R and NC 94022, and 140 RILs. The parents were sequenced at 20X coverage while the population was sequenced at 3–5Xcoverage for each RIL. In total, more than 50% of the sequence data were mapped on the A sub-genome and more than 60% of the sequences were mapped on the B sub-genome. Although the amount of data produced from each plant sample was different, the proportion of mapped reads on the respective genomes was similar for each of the individual plants (Supplementary Fig. S1). All the sequence reads mapped to the diploid reference genome were used for haplotype-based SNP calling (Fig. 3). Data were mapped on the reference genome in two different ways explained as following: first, the reads were mapped to the concatenated A- and B-genomes taken together; second the same data were mapped to individual A- and B-genomes separately. These two approaches were used to identify co-dominant and dominant SNPs, respectively. A total of 81,599 non-redundant SNPs (accounting from the concatenated and individual mapping) between the two parents were identified and used for downstream analysis.

When mapped onto the concatenated genome, a total of 40,106 polymorphic SNPs between the two parents were identified. Genotyping the RILs with these markers reduced the number of SNPs to 6,313, which were segregated in the RIL population. Further, considering the SNPs with less than 20% missing data, a total of 5,743 SNPs was identified as polymorphic and non-distorted in the population. We used a cut-off of 20% for missing data to retain significant number of SNPs without losing many SNP data points. At the same time, our criteria were stringent enough to use the SNP calls without having to impute the data.

In case of mapping the reads to individual A- and B-genomes, separately, a total of 52,677 (A-genome) and 16,072 (B-genome) SNPs identified between the two parents were used to genotype the RILs resulting in 7,894 (A-genome) and 6,016 (B-genome) markers to be present in the population, which ultimately resulted in identification of 5,343 and 3,885 SNPs with less than 20% missing data to be identified from the A- and the B-genomes, respectively. Therefore, in total, 5,743 SNPs and 9,228 SNPs were identified by mapping the sequence reads on both diploid A- and B- genomes (together) and either A- or B- genome (separate), respectively. Thus, a non-redundant set of 12,248 SNPs were identified, and 11,106 SNPs finally could be used in bin mapping-based linkage map with a map length of 2004 cM. Bin mapping was followed by QTL analysis (Fig. 3). The SNPs were distributed throughout the 20 LGs with the most SNPs occurring on chromosome A03 (1,348 SNPs) and the fewest on chromosome B10 (104 SNPs) (Table 2).

**Table 2:** Summary of total number of SNPs and bins constructed using the SNPs across 20 linkage groups.

Chr.	Number of SNPs	Number of bins	cM Length	Physical length (Mb)
A01	860	204	71.92	107.04
A02	559	362	207.69	93.87
A03	1348	402	85.89	135.06
A04	578	310	108.87	123.56
A05	1013	323	96.42	110.04
A06	596	309	151.99	112.75
A07	979	412	109.99	79.13
A08	312	291	112.57	49.46
A09	851	245	48.96	120.67
A10	307	152	54.37	109.46
B01	312	200	60.81	137.41
B02	549	322	133.12	109.00
B03	380	190	71.8	136.11
B04	356	247	89.65	133.62
B05	275	208	96.04	149.90
B06	404	337	157.58	137.15
B07	377	283	103.97	126.35
B08	574	571	98.91	129.61
B09	372	383	108.41	147.09
B10	104	65	35.06	136.18
**Total**	**11,106**	**5,816**	**2004.02**	**2383.4**

Number of SNPs represents the total number of polymorphic SNPs identified with the RIL population. Number of bins are the recombination bins identified within 300 Kb interval on the respective chromosomes based on the SNP markers used. The length in cM represents the genetic length of each linkage group constructed using the bins as markers. Physical length represents each chromosome size in terms of Mb.

### Recombination breakpoints and resolution of genes

**Figure 4:**
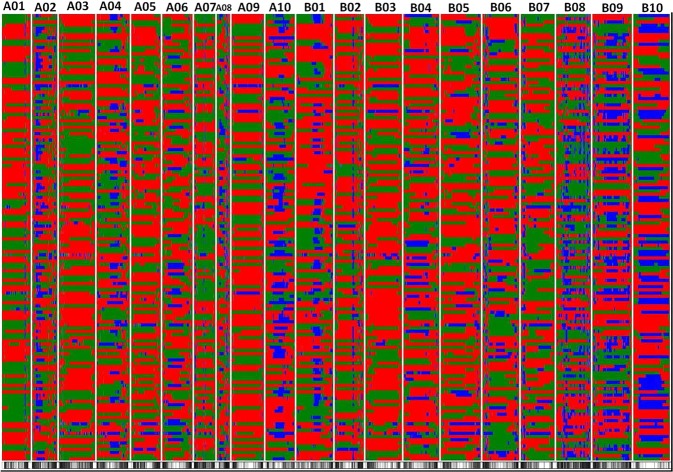
The recombination breakpoints identified in 140 recombinant inbred lines (RILs) using the non-distorted polymorphic SNPs. The chromosomes are labeled as A01 to A10 and B01 to B10 and are separated by vertical lines while each horizontal line represents a single RIL. Green and red bars represent segments from NC 94022 and SunOleic 97R genotypes, respectively. Blue bars represent the heterozygous/missing call. The black and white panel at the bottom indicates the consensus 5,816 bins identified in the entire RIL population.

**Figure 5:**
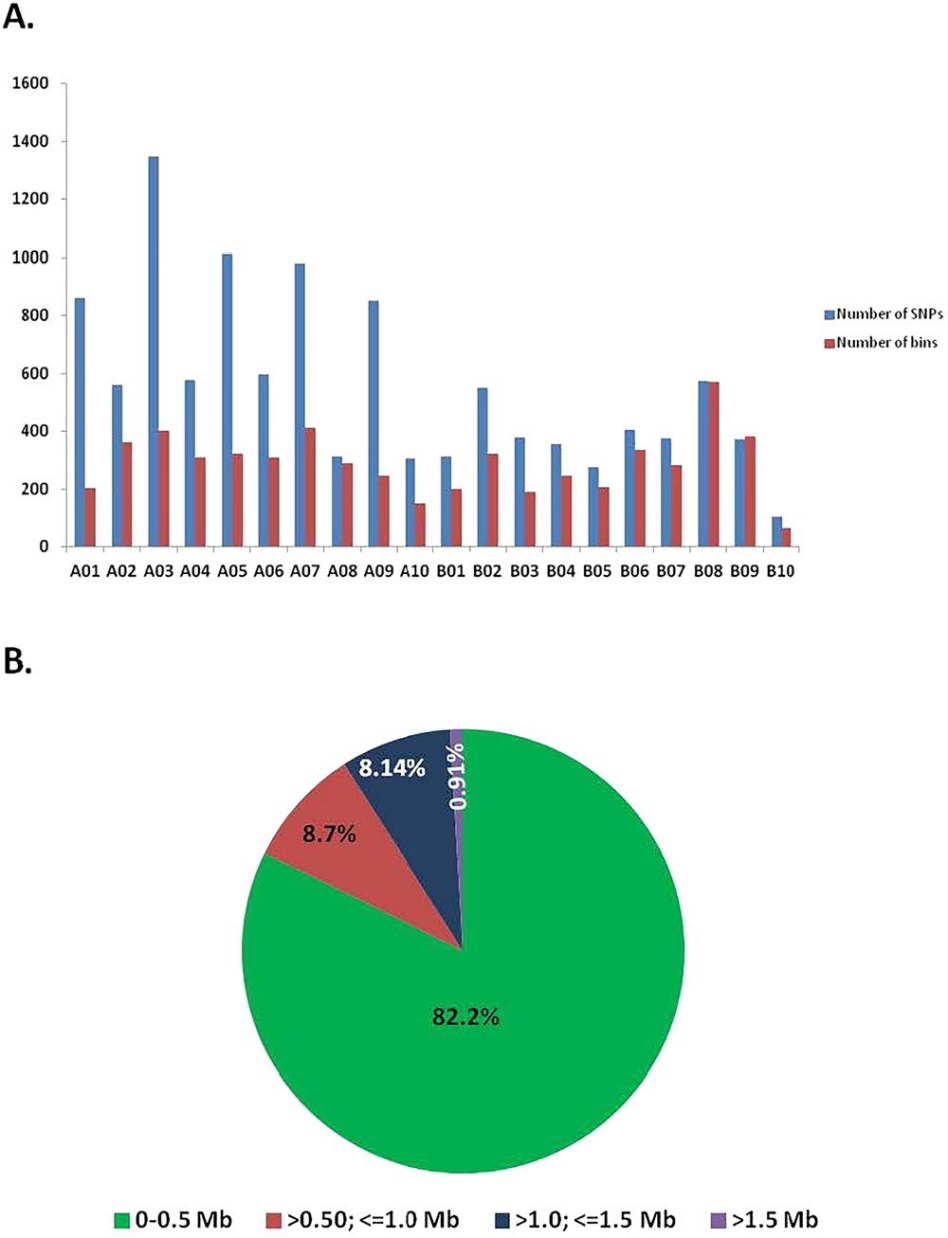
Features of recombination bin mapping in peanut. (**A**) Distribution of recombination bins and polymorphic SNPs within the RIL population identified on twenty peanut linkage groups (A01 to A10 and B01 to B10). Blue bars represent the number of SNPs and red bars represent the number of bins on respective LGs. Maximum number of SNPs were identified on LG A03, however, maximum number of bins were identified on B08. (**B**) Distribution of bin sizes identified in SunOleic 97R × NC 94022 population. More than 82% of the bins were of <= 0.5 Mb size indicating majority of recombination has been captured.

By using the sliding window approach on 11,106 SNPs segregating in 140 RILs, a total of 5,816 bins were identified (Fig. 4, Table 2). The minimum number of bins was identified on B10 with 65 and the maximum number of bins was identified on B08 with 571. The bin sizes were ranged from 6 bp to 32.7 Mb, with an average of 409.8 Kb and the median of 120.28 Kb. In total, 91% of bin size was less than one Mb while 9% bins (527 bins) had a size of more than one Mb (Fig. 5).

### Bin mapping-based QTL analysis

**Figure 6:**
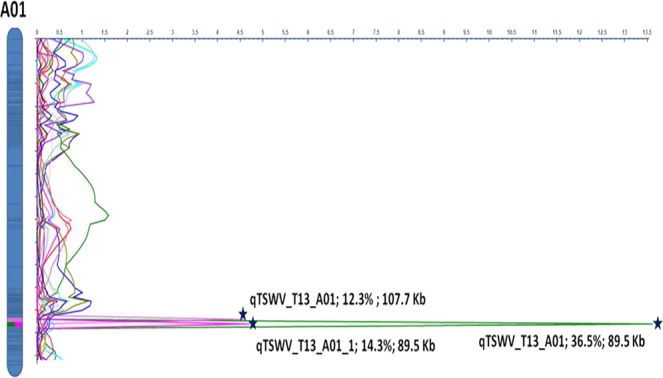
QTL map showing the major TSWV resistance QTL peaks at different LODs. Horizontal axis represents LOD values. Vertical bar represents the linkage group (LG) A01 with marker positions represented by horizontal black lines within them. A total of three major QTLs for Tomato spotted wilt virus (TSWV) are shown on LG A01. Stars are marked with the QTL name, PVE % and the distance covered on physical map. Colored horizontal lines within the LG bars represent the position of a major QTL.

Genotyping data of 5,816 bins were analysed together with phenotyping data collected for Tomato spotted wilt virus (TSWV) for two years out of four years but only three phenotyping data collections were significant (Fig. 1), one in 2011 and two collections in 2013 for both plantings. QTL analysis resulted in identification of three QTLs with up to 36.51% PVE (LOD values ranged from 4.49 to 13.50) (Table 3) for resistance to TSWV (Fig. 6). There were three major QTLs identified on LG A01 for 2011 and 2013 phenotype data, two QTLs (*qTSW_T13_A01* and *qTSWV_T13_A01_1*) were found to be flanked by bins (bin_1_9457148 and bin_1_9546698) encompassing an 89.5 Kb physical sequences (Fig. 6).

**Table 3:** Details of QTLs identified using bins as markers. Three QTLs for Tomato spotted wilt virus (TSWV) were identified on chromosome A01.

QTL^a^	Month	Pos. (cM)	LeftMarker	RightMarker	LOD	PVE^b^ (%)	Add^c^	Distance^d^ (kb)
*qTSWV_T13_A01*	July	63	bin_1_9457148	bin_1_9546698	13.5	36.51	−0.71	89.55
*qTSWV_T13_A01_1*	July	63	bin_1_9457148	bin_1_9546698	4.63	14.27	−0.39	89.55
*qTSWV_T11_A01*	August	62	bin_1_9018199	bin_1_9125906	4.49	12.37	−0.31	107.71

^a^*q* denotes qtl, T represent Tifton, GA; numbers 11 and 13 represent the years in which the phenotyping was done, alpha-numeric notations starting with A represent the chromosome number of A-genome.

*qTSWV_T13_A01_1* represent the QTL found for another replicate of TSWV phenotyping conducted during that year.

^b^Percent of phenotypic variation explained by the QTL.

^c^Additive effect of the QTL;

^d^Physical distance on the chromosome in terms of kilo-bases between the markers flanking the QTL.

### Candidate genomic region(s) for *Tomato spotted wilt virus* (TSWV)

The major QTL for TSWV with more than 36% PVE on LG A01flanked by bin_1_9457148 and bin_1_9546698 harbored 14 genes. These 14 genes included four chitinase family protein coding genes, two strictosidine synthase-1-like genes, and one gene each for leucine rich repeat receptor kinase, acetyl-coA-synthetase, cytochrome c oxidase assembly protein, tripeptydyl peptidase-2-like isoform and UV-radiation-resistance-associated-like protein coding gene, and three genes coded for unknown proteins. This 89.5 Kb region on chromosome A01contained three SNPs. One SNP was identified in the intergenic region and the other two were identified in the exons of two genes coding for acetyl-CoA synthetase and UV radiation resistance-associated-like protein. In order to scan the nearby regions of the identified 89.5 kb QTL, 100 kb flanking region was scanned to identify genes with SNPs. A total of seven SNPs in the flanking region of this QTL were found. One of these seven SNPs was identified within the exon of gene, *Aradu.75IX3* coding for serine/threonine-protein phosphatase; two SNPs were identified within the exon of *Aradu.XH3ZX* coding for regulator of Vps4 activity in the MVB pathway protein, and two SNPs were identified within the introns of genes *Aradu.9A16Q* and *Aradu.5D88D* coding for chitinase family protein and GDSL-like Lipase/Acylhydrolase family protein, respectively. Remaining two SNPs were present in an intron and an exon of *Aradu.X971L* (coding for an uncharacterized protein) (Supplementary Table S2).

### KASP marker development and validation

**Figure 7:**
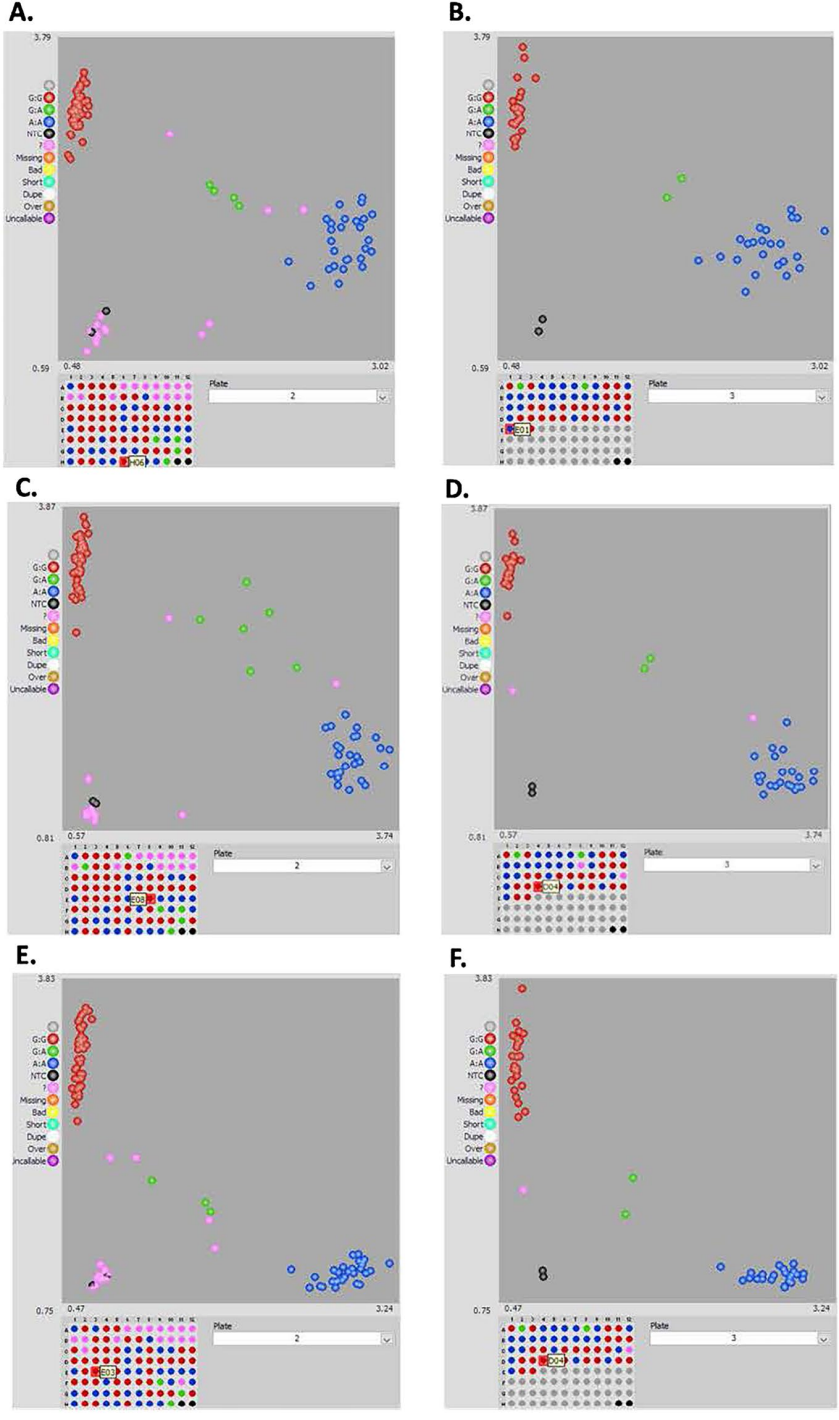
Representation of KASP genotyping assays using SNP markers flanking the QTL for resistance to TSWV on chromosome A01. Different types of validation of SNPs within or close to the bin marker flanking the major QTL. (**A,B**) shows KASP assay using SNP A01_9530252; (**C**,**D**) shows KASP assay of SNP A01_9604392; (**E**,**F**) shows KASP results of SNP A01_9192862 in the RIL population. The genotyping data for each type were viewed using the software of SNPviewer (LGC Genomics). The scatter plots for the X and Y axes were representing the alleles for a marker in the RIL population. The blue and red clusters were the homozygous alleles showing polymorphism.

**Figure 8:**
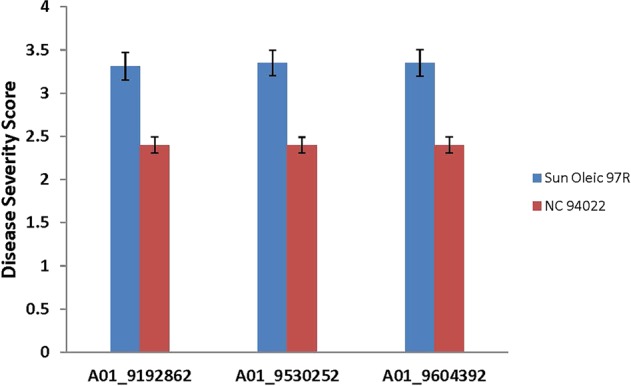
Showing the correlation between the average scores of diseases severity (phenotype) and the selected KASP markers of validated SNPs. Vertical axis was the average disease severity and horizontal axis was the markers. Unpaired t-test was conducted, and the p-values were estimated using the phenotyping data that the alleles came from SunOleic 97R (susceptible) or from NC 94022 (resistance). All three markers showed extremely statistically significant correlation with TSWV disease (p-value < 0.0001). The bars of error were the standard error calculated from mean values.

A total of three markers associated with TSWV QTL were used for developing robust KASP assays (Supplementary Table S3) for discriminating the parental genotypes and the resistant and susceptible RILs (Fig. 7). Out of the three TSWV associated markers, one marker (A01_9530252) was selected from within the QTL region flanked by bin_1_9457148 and bin_1_9546698, and other two markers (A01_9192862 and A01_9604392) were selected from flanking region of the QTL. A01_9192862 was identified as 264.2 Kb upstream and A01_9604392 was identified as 57.6 Kb downstream to the TSWV QTL on chromosome A01. All markers were successfully validated with 100% consistency in the parental genotypes between the *in silico* predicted SNPs and the allele calls obtained using KASP assays. In RIL population, consistency between *in silico* calls and the KASP assay was about 90% (Table 4). Overall, the KASP assay could clearly distinguish between the allelic variations in the population (Fig. 7). To study correlation between the phenotype and KASP genotype, we considered the resistant and susceptible lines based on their phenotype ratings. In terms of correlation with phenotyping data, SNP markers associated with TSWV, showed an average disease rating of 2.4 and 3.3 for RILs with alleles coming from NC94022 (R) and SunOleic 97 R (S), respectively (Fig. 8).

**Table 4:** SNP efficiency of markers validated using KASP assay.

	Concordant alleles^a^			Discordant alleles			Not validated	SNP efficiency (validation %)
	AA [S]	BB [R]	AB	AA [S]	BB [R]	AB		
A01_9192862	49	63	0	0	6	5	17	91.1
A01_9530252	46	68	0	1	5	6	14	90.5
A01_9604392	46	66	0	0	5	8	15	89.6

Concordant allele calls were the ones which confirmed the in-silico SNP call in the KASP validation. Discordant allele calls were the ones which showed mismatch of in-silico and KASP genotyping. Overall, the SNP efficiency was ~90% for each SNP. Some of the markers could not be assigned any genotype in KASP assay were therefore considered not validated.

^a^AA represent homozygous markers from the susceptible parent (Sun Oleic 97R); BB represents the homozygous markers from the resistant parent (NC 94022); AB represents the rare heterozygous call.

## Discussion

Genome-wide marker identification and polymorphism study in population are essential to underpin the quantitative and phenotypic traits related to the genetic architecture of a plant species. Breeding of peanut for traits like disease resistance has been a challenging task because of the narrow genetic diversity in the peanut germplasm^23^. As a result, in recent years, most of the QTL studies in peanut were conducted using a limited number of SSR and/or SNP markers. The turning-point was the availability of reference peanut genomes in 2016^3,12^ and now with a reference tetraploid genome^4^. There were several research groups reporting using high throughput NGS approach to conduct QTL studies and to identify the potential candidate genes and markers linked to the traits^15,16,24^. Recently, Agarwal *et al*.^15^ reported the first SNP based high-density genetic map using an improved WGRS based SNP identification approach^25^. Here we report the first bin-map in peanut with 5,816 bins in 20 linkage groups representing 11,106 SNP markers. The sequence reads were mapped to individual A- and B-genomes separately and to both the genomes taken together in order to identify two different kinds of SNPs. We observed that only 5,743 SNPs out of the total 40,106 parental SNPs were identified to be polymorphic in the RIL population when concatenated A- and B-genomes were used. Similarly, when mapping was done separately on individual A- and B-genomes, 5,343 out of 52,677 A-genome and 3,885 out of 16,072 B-genome parental SNPs were identified to be polymorphic in the RIL population. This observation that many SNPs identified between the parents could not be genotyped in the population can be attributed to low sequence coverage of RILs or false SNPs identified among the parents or very low level of polymorphism. False SNPs could be a result of the B-genome reads mapped to A-genome and vice-versa, thus detecting false SNPs against the background of A vs. B polymorphisms^26^. Such false SNPs were automatically omitted due to lack of polymorphism in the RIL population.

In this study, using WGRS approach, more than 11,000 SNPs were identified in RIL population, which represents the largest number of markers used for QTL mapping in peanut to date. Instead of using the SNPs as such for linkage mapping, a sliding window-based approach was used to identify bin markers where consecutive SNPs were merged into one bin (bin mapping). The total number of bins per RIL in peanut was about 131 bins (recombination breakpoints), which was higher than expected in comparison with other crops, such as in pepper (33), chickpea (32) and rice (33)^19,22,27^. In a study in maize, each RIL was estimated to have 3-5 recombination events on each chromosome^28^. However, in this study in peanut, 6–7 recombination events were identified on each chromosome (131/20 = 6.5). Peanut is an allotetraploid and is known to show to a meiotic behavior, where recombination happens between both homologus and homeologus chromosome pairs^29,30^. Such frequent tetrasomic recombination may have resulted in higher number of recombination events in peanut. The average bin size was ~410 Kb and more than 90% bins were less than 1 Mb, suggesting that majority of the recombination events were captured in this study. All 5,816 recombination bins were used as markers to construct this bin-based linkage map with 20 linkage groups and 2004 cM in length. Using this same RIL population, Khera *et al*.^10^ reported a linkage map using 248 SSR markers with a length of 1425.9 cM. In this study, bin-based marker number was increased by ~23 times. However, the map length was increased only by less than 600 cM. Bin mapping approach could reduce the number of potential false positive SNPs that might have been identified as a result of erroneous sequencing and also addresses the problem of handling a large set of markers by QTL analysis software^19,22^.

Linkage map constructed in this study using the bins defined out of SNPs covered a genetic distance of 2004 cM which is considerably less than the genetic length of over 3000 cM reported in a study by Agarwal *et al*.^15^ using WGRS approach. The reason could be attributed to the size of the population (141 RILs in the current study compared to 91 RILs), number and type of the markers used for map construction (5,816 bins containing 11,106 SNPs used in the current study instead of the 8,868 SNPs used). Also, the compact length of 2004 cM using 5,816 bins reflects the genotyping and bins construction accuracy. Errors in genotyping usually cause elongation of a linkage map. Therefore, reduced genetic map length in this study was probably caused by the variation in the number and type of the markers and change in the size of the mapping population.

This study identified an 89.5 Kb region on LG A01, which was associated with TSWV resistance. Same QTL for TSWV was also identified in earlier studies using the same RIL population. Khera *et al*.^10^ reported a QTL with a maximum of 29.1% PVE and Qin *et al*.^7^ also identified a similar TSWV QTL with 35.8%. In another recent study, 800 Kb QTL region for TSWV was identified in a F_6_ population obtained from a cross between Florida-EP ‘113’ and Georgia Valencia with 22.8% PVE and LOD value of 8.5^31,32^ also reported a major QTL for TSWV resistance. All these reported major QTLs were on linkage group A01, but none of these studies could resolve the potential candidate genes underlying the QTL. In this study we not only reported the QTL with a higher PVE of over 36% and LOD value of 13.5 but also fine mapped it to a less than 90 Kb region on chromosome A01. A total of 14 genes were identified in this region, and four out of the 14 genes coded for chitinase gene family proteins. Chitinases hydrolyse the β-1,4-linkages in chitin, an abundant N-acetyl-β-D-glucosamine polysaccharide which is a structural component of fungal cell walls and insect exoskeletons^33^. TSWV are transmitted to peanut by an insect vector called thrips that live, feed and reproduce on plant leaves and flowers. The cluster of chitinase genes identified in the QTL region seems to be the promising potential candidate genes that can be explored further to study host resistance to thrips and TSWV in peanut. Two strictosidine synthase-like genes were also identified within the QTL region. Strictosidine synthase-like gene has been involved in plant defense responses against cucumber mosaic virus in Arabidopsis^34^. Additionally, one LRR coding gene was identified on chromosome A01. Leucine-rich repeat receptor-like kinases (LRR-RLK) constitute a diverse group of proteins allowing the cell to recognize and respond to the extracellular environment. LRR family proteins are well known to play a role against diseases. For example, *Sw5* resistance gene that codes for CC-NB-LRR protein confers resistance to TSWV, and to other distinct tospoviruses, groundnut ringspot virus (GRSV) and tomato chlorotic spot virus (TCSV)^35–37^. Another example in tomato, modulation of LRR-RLK (SlSOBIR1) gene expression was shown to be triggered by secondary effects of the virus infection process^38^. Another gene was UV radiation resistance-associated-like protein coding gene identified in this study in the TSWV associated QTL region. Interestingly, UV-B (280–320 nm) radiation is known to act through signaling pathways, the components of which closely resemble those for pathogen resistance. UV stimulates transcription of genes important for defense-like pathogenesis-related proteins such as chitinase and *β*-1,3-glucanase^39^. A possibility of thrips response to be mediated via UV activated signaling pathway genes/proteins ultimately invoking chitinase and *β*-1,3-glucanase genes cannot be ruled out.

With our next objective to develop markers that can be implemented in breeding programs, we developed KASP markers for the major TSWV QTL. Three markers on chromosome A01 showed good segregation pattern in the RIL population. These three markers were found to be closely linked to the alleles transferred by the resistant (NC94022) and the susceptible (SunOleic 97R) parent in the population of 140 individual RILs and were able to predict the lines with alleles shared by resistant or susceptible parent with high accuracy. Accuracy rates of 91.1%, 90.5% and 89.6% for A01_9192862, A01_9530252, and A01_9604392, respectively, suggest the diagnostic nature of these markers. We developed KASP assays for as many as six SNPs identified within the TSWV QTL; however, only one marker could be amplified and showed polymorphism in the population. The other two markers were identified outside but in proximity to the bins flanking the QTL. In order to develop more markers closely linked to TSWV QTL more SNPs are needed to be identified within the 89.5 Kb region associated with the QTLs linked to the resistance to TSWV.

## Conclusions

We have developed the first peanut bin-map and identified amajor QTL on chromosome A01 linked to resistance to TSWV. The closely linked KASP markers could be used in breeding selection for resistant breeding lines. This study also demonstrated that bin-based linkage map and the QTLs analysis approach is effective in comparison with traditional QTL study, particularly, when the number of markers is too large to be accommodated on a genetic map. However, in order to pinpoint the gene(s) with high resolution, a large population will be needed to capture more recombination events (basis of increased resolution) which lacks in a bi-parental population. Approaches like association mapping and nested association mapping are being deployed in peanut to unravel the genes responsible for these resistance traits with high resolution^40^.

## Methods

### Plant material and phenotyping

Two peanut lines, SunOleic 97R and NC94022, were selected as parents to develop a RIL mapping population^10^. SunOleic 97R, the female parent is a runner market-type with high oleic acid content and is very susceptible to TSWV as a public released cultivar^41^. NC 94022, the male parent (Virginia type), has the highest resistance to TSWV as evaluated in the field studies^42^, which was a selection from a cross between N91026E, an early maturing Virginia type line moderately susceptible to TSWV, and PI 576638, a *hirsuta* botanical type line from Mexico^43^. Phenotyping in the field conditions for TSWV was conducted from 2010 to 2013 (four years) in Bellflower Farm of USDA-ARS, Crop Protection and Management Research Unit at Tifton, Georgia, each year with two planting dates (April and May) and in different fields as rotation with other crops of corn and cotton to insure higher disease incidences^10,11^. A randomized complete block design with at least three replications was used. Disease ratings for TSWV were scored on a scale of 1 to 10 disease intensity^44,45^ based on a visual rating on a whole plot basis exhibiting typical symptoms such as stunting, ringspot, leaf necrosis and chlorosis. The 1 to 10 scale represented a percentage of diseased plants with the typical symptoms (1, 1.5, 2, 3, 4, 5, 6, 7, 8, 9, 10 equals 0%, 1–10%, 11–20%, 21–30%, 31–40%, 41–50%, 51–60%, 61–70%, 71–80%, 81–90%, and 91–100%, respectively)^10^.

### Library construction and sequencing

Whole-genome shotgun sequencing strategy was used to construct the paired end libraries from the DNA extracted from peanut lines used in this study. Paired end sequencing libraries were sequenced using an Illumina HiSeq 2000 platform (Illumina, San Diego, CA, USA). Parental genotypes were sequenced separately at a high sequencing depth, ~20X for SunOleic 97R and NC94022 with insert size of 300 bp. Paired read data of 150 bp read length were generated for each of the two parents. Individual RILs were sequenced at ~3–5X coverage with (100 × 2) bp read length^15^. Filtered reads were used for alignment to the reference genome assemblies of *A. duranensis* (v1, www.peanutbase.org) and *A. ipaensis* (v1, www.peanutbase.org) separately and taken together used for SNP identification and genotyping^15,25^.

### SNP identification, genotyping and bin mapping

SNP identification was carried out using the novel haplotype-based SNP calling pipeline developed by Clevenger *et al*. (unpublished), which was an improved version of SWEEP as described^25^, as detailed methodology explained earlier^15^ and in Fig. 3. Read alignment for SNP calling was carried out in two different ways. The read data were aligned to both A- and B-genome individually and to the concatenated genomes in order to identify both co-dominant and dominant SNPs^15,25^. Further, a non-redundant set of SNPs identified by two approaches was considered for further analysis. All 11,106 SNPs were then used for defining bins containing SNPs. A parent-dependent 15 bp sliding window approach was used to identify true recombination breakpoints. This was determined based on the ratio of the alleles in the sliding window using a perl script^27^. A total of 11,106 SNPs identified were scored as “A” and “B” for alleles from the parents, NC94022 and SunOleic 97R, respectively, and for each RIL line. Windows with twelve or more alleles from one parent were considered as homozygous for that respective genome region. The perl script was used according to peanut genome co-ordinates with needed changes. For each RIL, the A and B alleles ratio within the window was counted. The definition of a recombination break point was the transition from one to another genotype. The bins were recombination breakpoints, obtained from all RIL lins, aligned and compared over 300 Kb intervals. For each individual RIL, successive bins in 300 Kb region were merged if there were no recombination events observed. The successive intervals without recombination break points in the population were combined as a single bin. Then, these bins were used as markers to construct genetic linkage map.

### Linkage map construction and QTL identification

Filtered and high quality SNPs with less than 20% missing were used for construction of bin maps^15^. Bins constructed thus were used as markers for genetic mapping using QTL IciMapping v4.1^46^. Bins were grouped at LOD ≥ 5 and ordered using the nnTwoOptalgorithim. Kosambi’s mapping function was used in order to convert recombination frequency into map distance in centiMorgan (cM). The order was confirmed using the ripple command. The genotyping and phenotyping data were used for QTL identification using inclusive composite interval mapping (ICIM) function, QTL IciMapping v4.1^46^ and a LOD ≥ 3. A QTL was considered as a major QTL only with a PVE explained >10%.

### KASP assay development

Detailed KASP assay was used as reported by Agarwal *et al*.^15^, and briefly described as follow. Sequences of SNP markers flanking QTL for TSWV on chromosome A01 were converted to KASP markers. The KASP genotyping assay is fluorescence (FRET) based assay that enables identification of biallelic SNPs^15^. Two allele-specific forward primers along with tail sequences and one common reverse primer were synthesized by LGC Genomics (http://www.lgcgroup.com) (Supplementary Table S3). The reaction mixture was prepared following the manufacturer’s instructions with minor modifications in number of cycles (KBioscience; http://www.lgcgroup.com/products/kasp-genotypingchemistry/#.VsZK7PkrKM8). Briefly, KASP assays^15^ were run with 10 µL final reaction volume containing 5 µL KASP master mix, 0.14 µL primer mix, 2 µL of 10–20 ng/µL genomic DNA, and 2.86 µL of water. The following thermal cycling conditions were used: 15 min at 95 °C followed by 10 touchdown cycles of 20 s at 94 °C and 1 min at 61–55 °C (dropping 0.6 °C per cycle), and then 26 cycles of 20 s at 94 °C and 1 min at 55 °C. For each assay 26 cycles were used. The fluorescent endpoint genotyping method was carried out using Roche Light Cycler 480-II instrument (Roche Applied Sciences, Indianapolis, IN, USA).

## Supplementary information

Supplementary information

## Data Availability

All data generated or analyzed during this study are included in this articleand the additional files. The raw WGRS data will be deposited in peanut database (https://www.peanutbase.org) after completion of other ongoing analyses.
