# Publisher Correction: A recombination bin-map identified a major QTL for resistance to Tomato Spotted Wilt Virus in peanut (*Arachis hypogaea*)

**DOI:** 10.1038/s41598-020-58891-x

**Published:** 2020-02-05

**Authors:** Gaurav Agarwal, Josh Clevenger, Sandip M. Kale, Hui Wang, Manish K. Pandey, Divya Choudhary, Mei Yuan, Xingjun Wang, Albert K. Culbreath, C. Corley Holbrook, Xin Liu, Rajeev K. Varshney, Baozhu Guo

**Affiliations:** 1 USDA-ARS, Crop Protection and Management Research Unit, Tifton, GA USA; 2 University of Georgia, Department of Plant Pathology, Tifton, GA USA; 3 International Crops Research Institute for the Semi-Arid Tropics (ICRISAT), Center of Excellence in Genomics & Systems Biology, Hyderabad, India; 4 Mars Wrigley Confectionery, Center for Applied Genetic Technologies, Athens, GA USA; 5 University of Georgia, Center for Applied Genetic Technologies, Athens, GA USA; 6 Shandong Academy of Agricultural Sciences, Peanut Research Institute, Qingdao, China; 7 Shandong Academy of Agricultural Sciences, Biotechnology Research Center, Jinan, China; 8 USDA-ARS, Crop Genetics and Breeding Research Unit, Tifton, GA USA; 9 BGI-Shenzhen, Shenzhen, China; 10 Present Address: The Leibniz Institute of Plant Genetics and Crop Plant Research (IPK), Gatersleben, Germany

Correction to: *Scientific Reports* 10.1038/s41598-019-54747-1, published online 03 December 2019

The original PDF version of this Article contained an error in the Abstract.

“Toma to spotted wilt virus (TSWV) is a devastating disease to peanut growers in the South-eastern region of the United States.”

now reads:

“Tomato spotted wilt virus (TSWV) is a devastating disease to peanut growers in the South-eastern region of the United States.”

This error has now been corrected in the PDF version of the Article. The HTML version was correct at time of publication.

