# Supplementary material for: A recombination bin-map identified a major QTL for resistance to Tomato Spotted Wilt Virus in peanut (Arachis hypogaea)
Source: Sci Rep. 2019 Dec 3;9:18246. doi: 10.1038/s41598-019-54747-1 (PMC6890646; doi:10.1038/s41598-019-54747-1)
Supplement: Supplementary file 1 — Supplementary information [file 41598_2019_54747_MOESM1_ESM.pdf]

Supplementary Table S1. Phenotypic variation of Tomato spotted wilt virus (TSWV) diseases in S-population RILs. Disease severity was measured on a scale of 1 to 10 with 1 signifying the high degree of resistance and 10 signifying the high susceptibility to diseases.

**Trait_month_day_year**	**SunOleic 97R**	**NC 94022**	**Mean**	**SD***	**CV# (%)**	**Min**	**Max**
TSWV_2011	3	1.3	2.4	0.9	37	1	5
TSWV_7_24_2013	4.7	1.7	2.8	1	37.1	1.3	5
TSWV_7_24_2013_1	3.3	1.3	2.8	1.2	43.2	1	4

*SD = standard deviation
#CV = coefficient of variation expressed as percentage

Supplementary Table S2. Details of major QTLs identified for Tomato spotted wilt virus (TSWV). Genes and SNPs with in the major QTLs identified for TSWV, on chromosome A01 listed. 100 Kb flanking (up- and down-stream) to the QTLs were also scanned to identify the SNPs and genes present surrounding the identified QTLs.

QTL interval on physical map	Gene name	Chromosome:gene locus	SNP position	Exon/ Intron	SNP (NC 94022/Sun Oleic 97R)	Gene function
bin_1_9457148 - bin_1_9546698	Aradu.R403Z	A01:9459385:9461584	9459521	Exon	T/C	Acetyl-CoA synthetase
	Aradu.174XF	A01:9461623:9464072	_	_	_	Unknown protein
	Aradu.N0ICS	A01:9460399:9465094	_	_	_	Receptor-like protein kinase (Leucine-rich repeat)
	Aradu.HA9JS	A01:9472535:9479133	_	_	_	Strictosidine synthase 1-like
	Aradu.R03U5	A01:9480229:9482635	_	_	_	Cytochrome C oxidase assembly protein
	Aradu.A2Q98	A01:9466297:9468320	_	_	_	Strictosidine synthase 1-like
	Aradu.Y2B0V	A01:9512238:9512927	_	_	_	Unknown protein
	Aradu.118NW	A01:9504106:9506604	_	_	_	Chitinase family protein
	Aradu.A2QA1	A01:9483590:9488632	_	_	_	Chitinase family protein
	Aradu.Q1JIJ	A01:9496202:9499423	–	–	–	Tripeptidyl-peptidase 2-like isoform X1
	Aradu.JL35E	A01:9526513:9530482	9530252	Exon	A/G	UV radiation resistance-associated-like protein
	Aradu.W9SJN	A01:9522348:9524790	_	_	_	Chitinase family protein
	Aradu.8N0H3	A01:9533696:9534990	_	_	_	Chitinase family protein
	Aradu.G6HPF	A01:9519501:9520406	_	_	_	Unknown protein
100 Kb flanking to bin_1_9457148 - bin_1_9546698	Aradu.9AI6Q	A01:9562382:9564402	9563144	Intron	C/T	Chitinase family protein
	Aradu.XH3ZX	A01:9589707:9592075	9589841	Exon	T/C	Regulator of Vps4 activity in the MVB pathway protein
	Aradu.XH3ZX	A01:9589707:9592075	9589926	Exon	T/G	Regulator of Vps4 activity in the MVB pathway protein
	Aradu.75IX3	A01:9646248:9649054	9647526	Exon	C/T	serine/threonine-protein phosphatase 7 long form homolog
	Aradu.5D88D	A01:9594974:9602683	9599954	Intron	T/C	GDSL-like Lipase/Acylhydrolase superfamily protein
	Aradu.X971L	A01:9574283:9586995	9574939	Exon	A/T	Uncharacterized protein
	Aradu.X971L	A01:9574283:9586995	9585154	Intron	A/G	Uncharacterized protein

Supplementary Table S3. List of primer sequences for KASP assay of SNPs developed and validated for tomato spotted wilt virus (TSWV).

ID	Primer_Allele FAM	Primer_Allele HEX	Primer_Common	Allele FAM	Allele HEX
A01_9192862	GTACTTAATGGATGGGAGGAATCGA	ACTTAATGGATGGGAGGAATCGG	TTGCATCTTGAAGTATCTGTGAGTCAGAT	A	G
A01_9604392	ATAAATTATAGTTGGCTCGTTGCCAATATA	AATTATAGTTGGCTCGTTGCCAATATG	CCTAATCCCGATTCCCAAATTTCCAATAT	A	G
A01_9530252	CAGCTTTTGAACATGCTGTCCTCTA	AGCTTTTGAACATGCTGTCCTCTG	CATCTGGCTAAAGTATGTTCTTGTGTTCTT	A	G

A.

B.

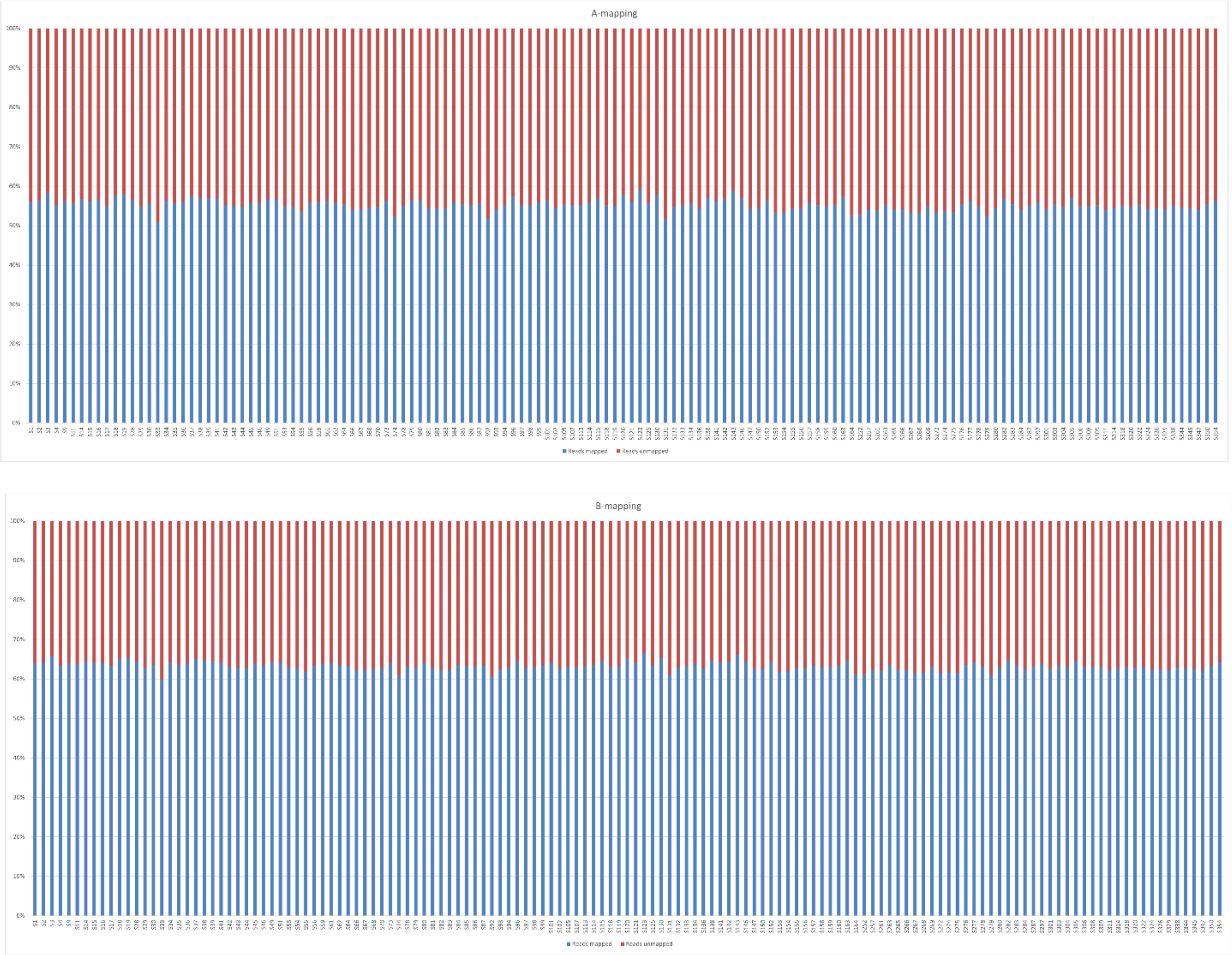

Figure S1. Mapping of reads on A- and B-genomes of peanut: reads mapped in blue and unmapped in red.
